# Hydrophilic intraocular lens opacification after posterior lamellar keratoplasty - a material analysis with special reference to optical quality assessment

**DOI:** 10.1186/s12886-017-0546-8

**Published:** 2017-08-22

**Authors:** Bert C. Giers, Tamer Tandogan, Gerd U. Auffarth, Chul Y. Choi, Florian N. Auerbach, Saadettin Sel, Christian Mayer, Ramin Khoramnia

**Affiliations:** 1 International Vision Correction Research Centre and the David J. Apple International Laboratory for Ocular Pathology, Department of Ophthalmology, University of Heidelberg, INF 400, 69120 Heidelberg, Germany; 2 Department of Ophthalmology, Kangbuk Samsung Hospital, Sungkyunkwan University School of Medicine, Seoul, South Korea; 3 Eye Clinic, Klinikum rechts der Isar der Technischen Universität München, Munich, Germany

**Keywords:** Cornea, Fuchs endothelial dystrophy, Posterior lamellar keratoplasty, Descemet membrane endothelial keratoplasty, Cataract surgery, Intraocular lens, Optical quality, Complications, IOL explantation

## Abstract

**Background:**

Laboratory analysis and optical quality assessment of explanted hydrophilic intraocular lenses (IOLs) with clinically significant opacification after posterior lamellar keratoplasty (DMEK and DSAEK).

**Methods:**

Thirteen opacified IOLs after posterior lamellar keratoplasty, 8 after descemet stripping automated endothelial keratoplasty (DSAEK), 3 after descemet membrane endothelial keratoplasty (DMEK) and 2 after both DSAEK and DMEK were analysed in our laboratory. Analyses included optical bench assessment for optical quality, light microscopy, scanning electron microscopy (SEM) and energy dispersive X-Ray spectroscopy (EDS).

**Results:**

In all IOLs the opacification was caused by a thin layer of calciumphosphate that had accumulated underneath the anterior optical surface of the IOLs in the area spared by the pupil/anterior capsulorhexis. The calcifications lead to a significant deterioration of the modulation transfer function across all spatial frequencies of the affected IOLs.

**Conclusions:**

The instillation of exogenous material such as air or gas into the anterior chamber increases the risk for opacification of hydrophilic IOLs irrespective of the manufacturer or the exact composition of the hydrophilic lens material. It is recommended to avoid the use of hydrophilic acrylic IOLs in patients with endothelial dystrophy that will likely require procedures involving the intracameral instillation of air or gas, such as DMEK or DS(A)EK.

## Background

The opacification of hydrophilic acrylic intraocular lenses (IOLs) is a rare complication, usually occurring in the late postoperative period [1]. The exact causes and pathomechanisms leading to hydrophilic IOL opacification are unknown. There have been sporadic reports about high incidences of IOL opacification affecting whole batches of IOLs of individual manufacturers irrespective of secondary surgical interventions or comorbidities [2–4], so that material impurities and faulty manufacturing or storage as well as interactions with the packaging material have been suggested as causative factors in these cases. Individual factors, such as ocular inflammation or ocular and systemic comorbidities that affect ocular metabolism, may contribute to the process [2, 3].

Secondary surgical interventions with instillation of foreign material into the anterior chamber, such as air, gas or rtPA [5] seem to increase the risk of IOL opacification. In particular, there has been an increasing number of reports since 2011/2012 about hydrophilic IOL opacification following posterior lamellar keratoplastic surgery involving the intracameral instillation of air or gas [6–13]. In all of these cases, the opacification was limited to a more or less circular area of the anterior optical surface, corresponding to the zone of contact with the instilled air or gas.

Opacifications of the IOL optic may cause reduced visual acuity, decreased contrast sensitivity and glare [3]. In clinically significant IOL opacifications an IOL explantation is the only treatment option. IOL explantation procedures are, however, often associated with an increased complication rate [14].

We report on the laboratory examination of 13 opacified IOLs which were explanted due to reduced visual acuity after posterior lamellar keratoplasty. All IOLs were examined using light microscopy, scanning electron microscopy and X-ray spectroscopy. In those specimen, that were explanted in toto, the optical properties were examined at an optical bench using modulation transfer function (MTF) measurements and documenting United States Air Force (USAF) 1951 resolution target images. To our knowledge, this is the largest series of opacified IOL explants, that systematically analyses not only the nature of the IOL opacification using light and electron microscopy as well as elementary analysis, but adds an analysis of the explanted IOLs' optical properties using an optical bench. It therefore provides an objective measure to the clinical findings of visual deterioration and further adds to our understanding of IOL opacification after posterior lamellar keratoplasty procedures.

## Methods

### IOL specifications, handling and gross examination

A total of 13 IOLs that had been explanted due to clinically significant opacification after posterior lamellar keratoplasty procedures were examined at the David J Apple International Laboratory for Ocular Pathology at the Department of Ophthalmology of the University of Heidelberg. In 8 subjects the opacification had appeared after descemet stripping automated endothelial keratoplasty (DSAEK; IOLs 1–5, 7, 8, 10) and 3 subjects had a descemet membrane endothelial keratoplasty (DMEK; IOLs 11–13) performed prior to the occurrence of IOL opacification. In two cases (IOLs 6 and 9), the patients had first received a DMEK and in the later course a DSAEK in the same eye prior to IOL opacification. IOL specifications, patient data and clinical history (as far as available) are summarized in Table 1. For shipment, the explanted IOLs were submerged in isotonic sodium-chloride solution. After gross macroscopic inspection, the IOLs were examined using an Olympus BX50 light microscope (Olympus Optical Co. Ltd., Tokyo, Japan). Photos were acquired using an Olympus C-7070 Camera (Olympus Optical Co. Ltd., Tokyo, Japan).

**Table 1:** IOL specifications and clinical history

IOL no.	IOL type (manufacturer)	IOL design, material (water content)	Age at implantation/sex	Implantation	Other procedures	Opacification noticed	Explantation	Associated conditions
1	Akreos Adapt AO (Bausch & Lomb)	One-piece, hydrophilic acrylic (26%)	55/M	n.k.	DSAEK	6 months after DSAEK	2011	Fuchs endothelial dystrophy
2	620 H (Rayner)	One-piece, HEMA/MMA (26%)	91/M	Aug 2007	DSAEK Feb 2011, Rebubbling	Sep 2011	n.k.	Fuchs endothelial dystrophy
3	Centerflex 970 H (Rayner)	One-piece, HEMA/MMA (26%)	79/F	Apr 2007	DSAEK Nov 2009	Jul 2011	Jul 2012	Fuchs endothelial dystrophy
4	620 H (Rayner)	One-piece, HEMA/MMA (26%)	77/M	Mar 2009	DSAEK Aug 2009 and Repeat-DSAEK Oct 2010	Oct 2011	Mar 2012	Fuchs endothelial dystrophy Retinal detachment surgery 1997 (external plomb)
5	Sulcoflex Toric 653 T (Rayner)	One-piece, HEMA/MMA (26%)	68/F	Dec 2010 (as piggyback sulcus IOL)	DSAEK Sep 2011	Sep 2012	n.k.	Primary Akreos IOL (Bausch & Lomb) also opacified
6	620 H (Rayner)	One-piece, HEMA/MMA (26%)	n.k./M	Feb 2008	DSAEK Jul 2012, DMEK Mar 2012, Re-Bubbling Mar 2012	Sep 2012	Oct 2012	Fuchs endothelial dystrophy, Myopia
7	Superflex asph. 920H (Rayner)	One-piece, HEMA/MMA (26%)	n.k./M	Aug 2010	DSAEK May 2011 Repeat DSAEK Oct 2011	Mar 2013	Aug 2013	Herpes simplex keratitis
8	n.k. (Rayner)	One-piece, hydrophilic acrylic	n.k.	2006	DSAEK 2010 Penetrating Keratoplasty 2011	Jun 2012	Jan 2014	n.k.
9	Superflex asph. 920H (Rayner)	One-piece, HEMA/MMA (26%)	63/F	Jul 2008	DMEK Aug 2013 + Rebubbling DSAEK Nov 2013	May 2014	n.k. (received in Sep 2014)	Fuchs endothelial dystrophy, bullous keratopathy
10	C-flex asph. (Rayner)	One-piece, HEMA/MMA (26%)	n.k./M	2012	DSAEK 2012 DSAEK 2014	Between the two DSAEK procedures	Dec 2014	Glaucoma surgery in the same eye before 2012
11	Acri.Lyc 44S-5 (Acri.Tec)	Plate haptic, hydrophilic acrylic (25%) with hydrophobic surface	77/F	Sep 2003	DMEK 2012 Penetrating Keratoplasty 2013	late 2013	Nov 2014	Fuchs endothelial dystrophy
12	C-flex 570C (Rayner)	One-piece, HEMA/MMA (26%)	69/F	Jun 2006	DMEK Aug 2012	Oct 2013	Jan 2015	Glaucoma
13	CT Asphina 409 M (Zeiss)	Plate haptic, hydrophilic acrylic (25%) with hydrophobic surface	64/M	Feb 2013	DMEK Feb 2013 (combined procedure) + 2× Rebubbling	n.k.	Jan 2016	Fuchs endothelial dystrophy

*HEMA* hydroxyl-ethyl-meth-acrylate, *MMA* methyl-meth-acrylate, *n*.*k*. not known

### Assessment of optical quality

IOLs that were explanted in toto were tested using an optical bench (OptiSpheric IOL Pro, Trioptics, Germany) for optical quality according to a protocol described in ISO norm 11–979-2. Optical quality parameters assessed included the modulation transfer function (MTF), which describes the resolution of an optical system as the ratio of relative image contrast to object contrast. The optical bench features different types of targets as objects which are projected to the infinity through a collimator. The tested lens therefore creates an image of the target object at its focal plane. The light source illuminating the target is a broad band visible spectrum light source associated with a narrow band interferential filter at 546 nm. The measurement head composed of a microscope objective and an imaging system conjugated with a CCD camera scans through the imaging zone to find the best focus image created by the tested IOL. The microscope objective relays the image obtained via the IOL to the CCD, and the system’s software analyises the output of the CCD to create the MTF curve. For visualisation purposes United States Air Force (USAF) 1951 resolution targets were used to compare the image quality at the focal plane.

### Light microscopy

After optical measurement, all IOLs were split in halves and one half was analysed by light microscopy using the Alizarin Red and von Kossa stainings. For Alizarin Red staining, the IOL was fixated for two minutes in 4% buffered formaldehyde solution and rinsed with distilled water. Thereafter, the IOLs were submerged in 1% Alizarin Red solution for 3 min, rinsed again and were then examined using a light microscope. The specimen were then dehydrated and embedded in paraffin. Five micrometers vertical sections through the optical center of the IOLs were taken and subsequently stained using the von Kossa method. Sections were deparaffinized, rehydrated and incubated in 5% silver nitrate, treated with UV light for 30 min and rinsed several times. After incubation with 5% sodiumthiosulfate and a final rinsing step, sections were analysed using an Olympus BX50 light microscope equipped with a camera (Olympus C-7070, Olympus Optical Co. Ltd., Tokyo, Japan).

### Scanning electron microscopy and energy dispersive X-ray spectroscopy

The second halves of the explanted IOLs were sent to the Max-Planck-Institute for Polymer Research in Mainz, Germany, for further analysis including scanning electron microscopy (SEM) and energy dispersive X-Ray spectroscopy (EDS). For SEM analysis 2.5 μm cross sections through the IOL material were acquired using an ultramicrotome (UCT, Leica, Germany) and a 35° diamond knife (Diatome, Switzerland) and mounted on silicium grids. SEM examinations were carried out in low voltage (<1 kV) conditions using an SU8000 microscope (Hitachi, Japan). For a local chemical analysis EDS was performed using a Quantax 400 EDS detector (Bruker, Germany) to detect exogenous chemical elements within the IOL material.

This study solely involves laboratory analyses of IOL explants. No additional procedures on humans or animals were performed. An informed consent and ethics committee approval were therefore not required.

## Results

**Fig. 1:**
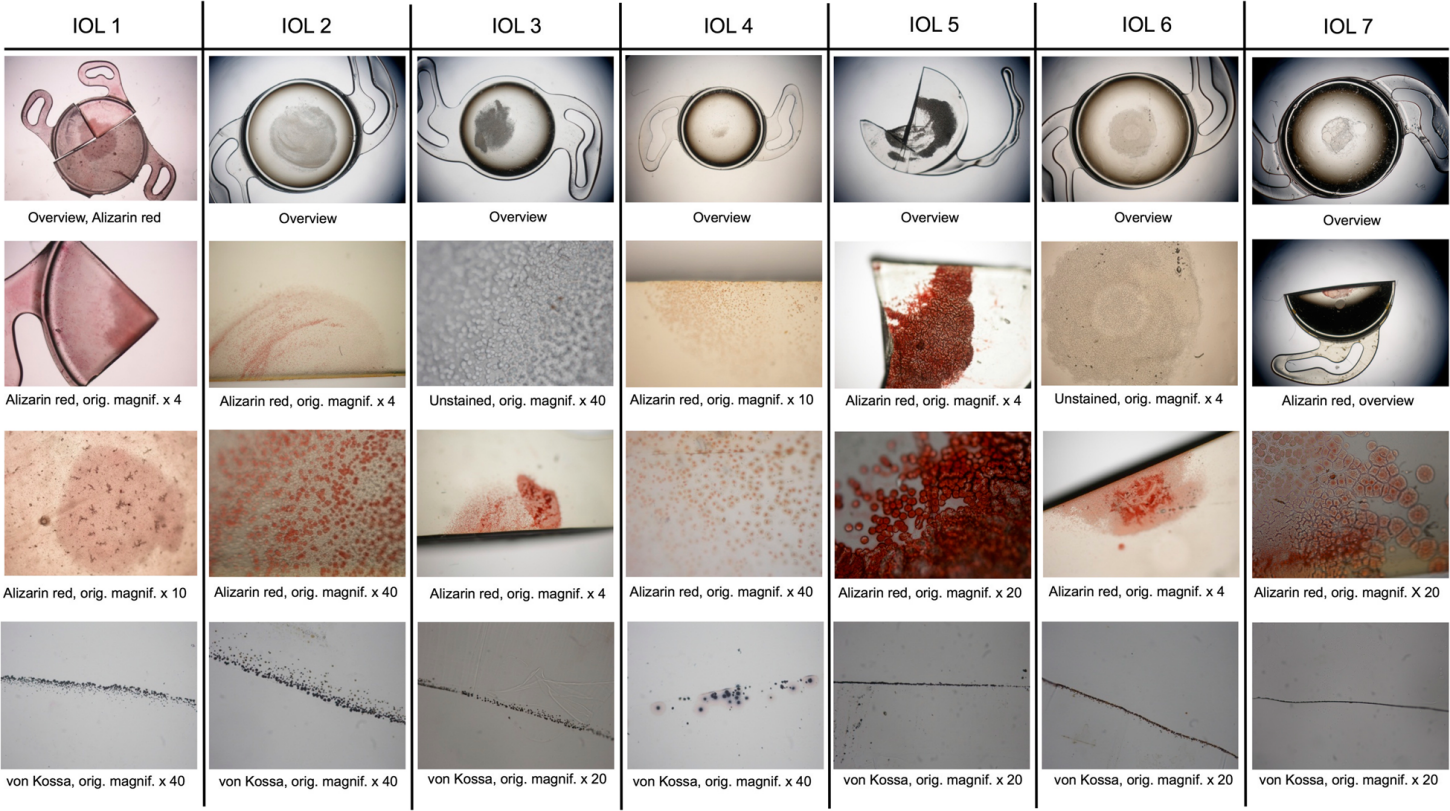
IOLs 1–7. Light microscopy images representing an overview of the IOL optic and higher magnification images with Alizarin Red and von Kossa stainings. Details and magnification as noted under each individual panel

**Fig. 2:**
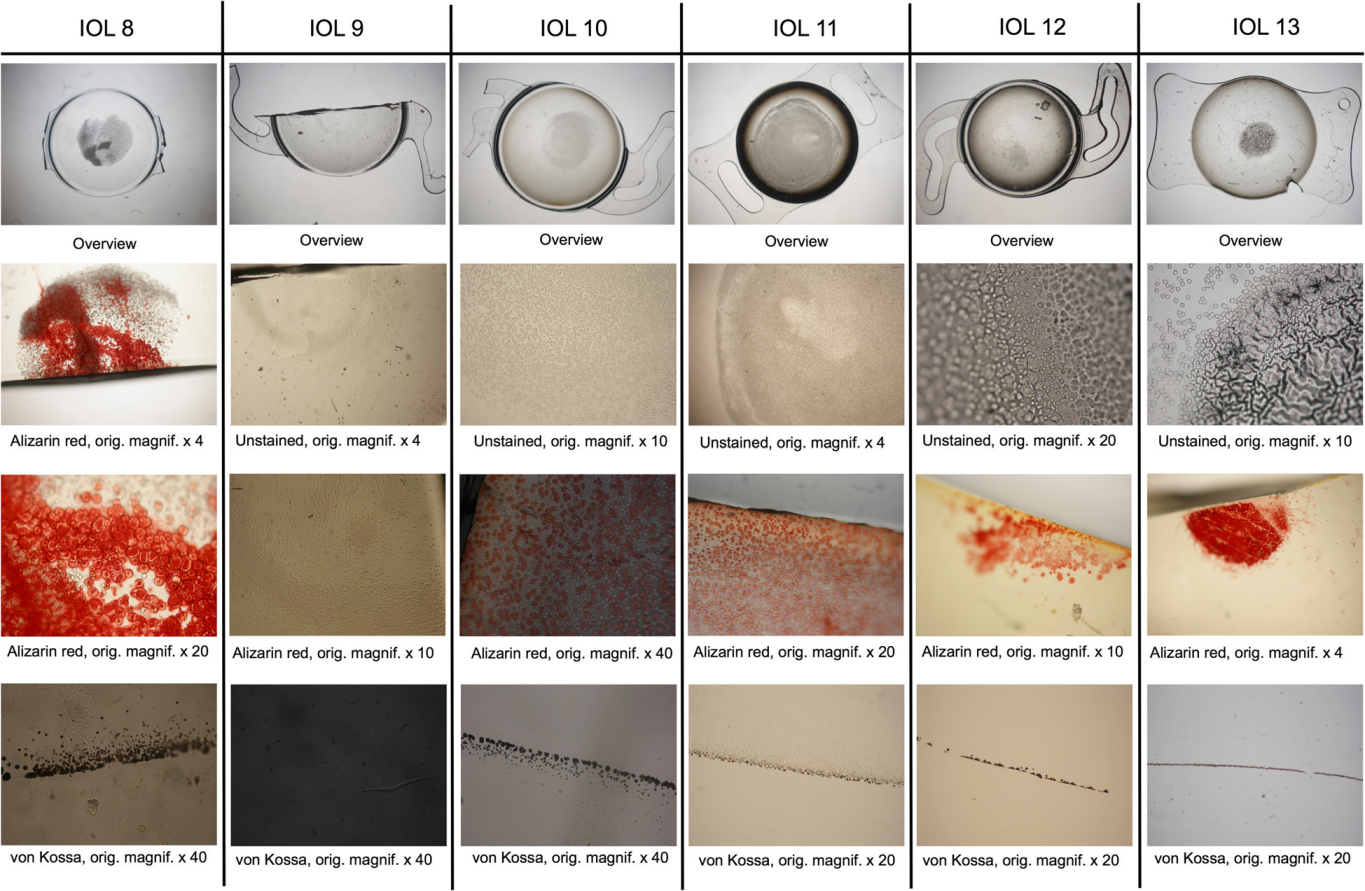
IOLs 8–13. Light microscopy images representing an overview of the IOL optic and higher magnification images with Alizarin Red and von Kossa stainings. Details and magnification as noted under each individual panel

Macroscopically, all IOLs showed a more or less circular opacification of the central anterior optical surface, sparing the peripheral optical zone and the haptics (Figs. 1 and 2). Light microscopy revealed numerous and sometimes confluent ovaloid or spherical deposits just underneath the anterior optical surface. The opacifications invariably stained positive with Alizarin Red for calcium. The von Kossa staining of cross sections of the IOLs revealed numerous fine granular crystalline deposits distributed in a line parallel to and immediately underneath the anterior optical surface of all IOLs. The posterior surfaces as well as the haptics of all examined IOLs were free of deposits.

**Fig. 3:**
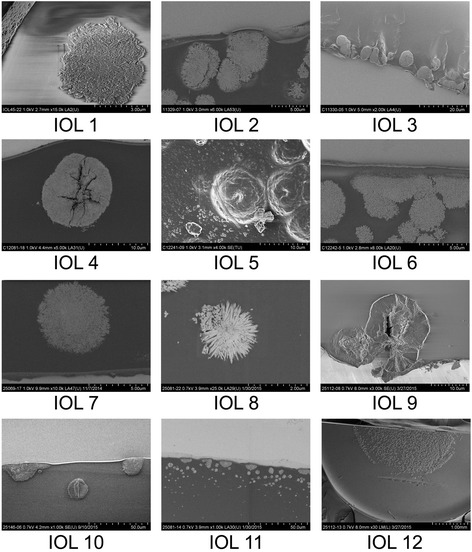
SEM-scans from cross-sections through the anterior optical surface (IOL 1–4 and 6–11), high magnification scan of the opacified anterior optical surface showing individual calcium deposits (IOL 5), low magnification overview of an IOL specimen showing the opacifications in the center of the anterior optical surface (IOL 12). Magnification and scale bar given in each individual panel

**Fig. 4:**
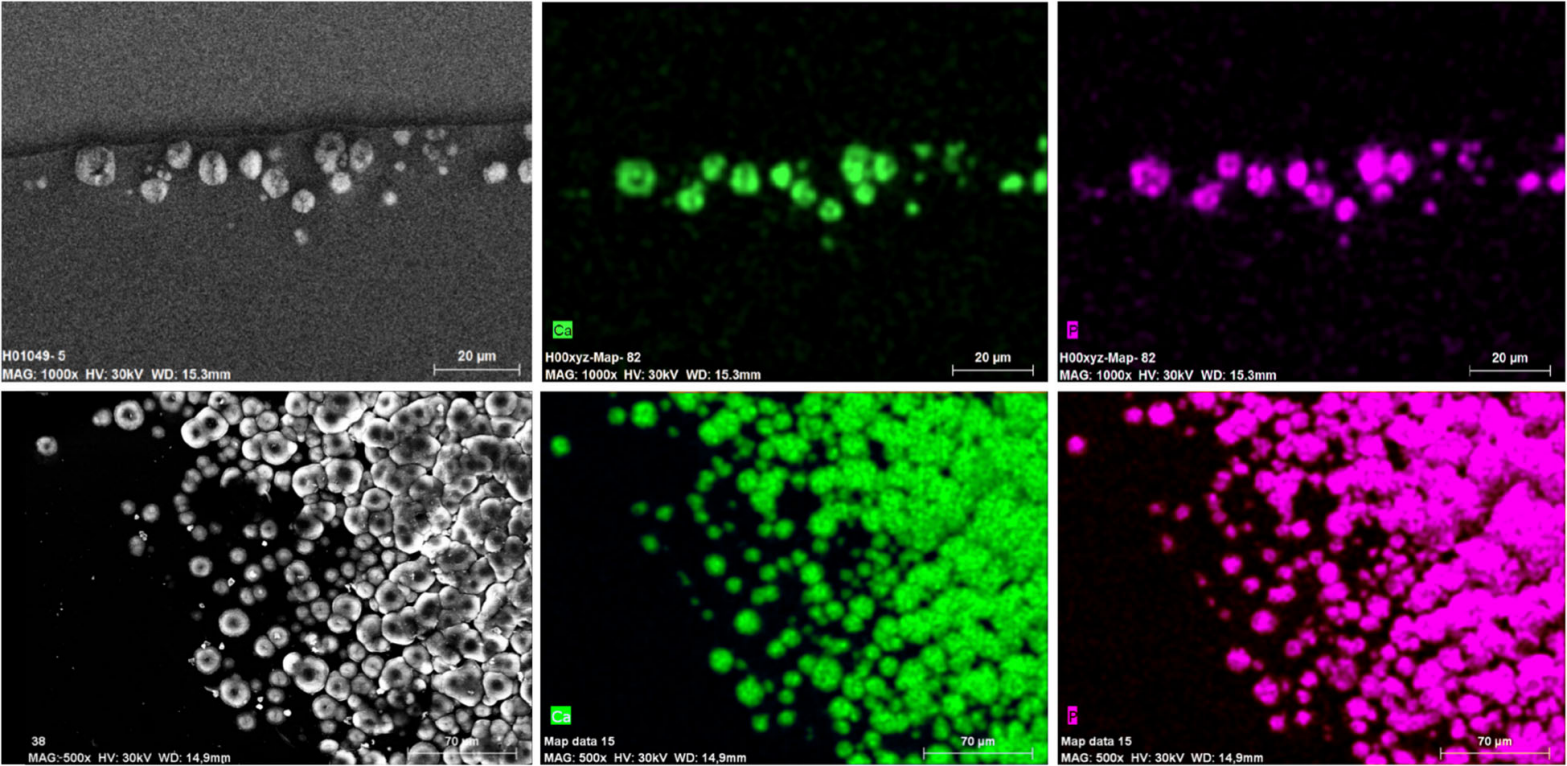
Exemplary Element Mapping showing calcium (green) and phosphorus (magenta) within the deposits in IOL 5

**Fig. 5:**
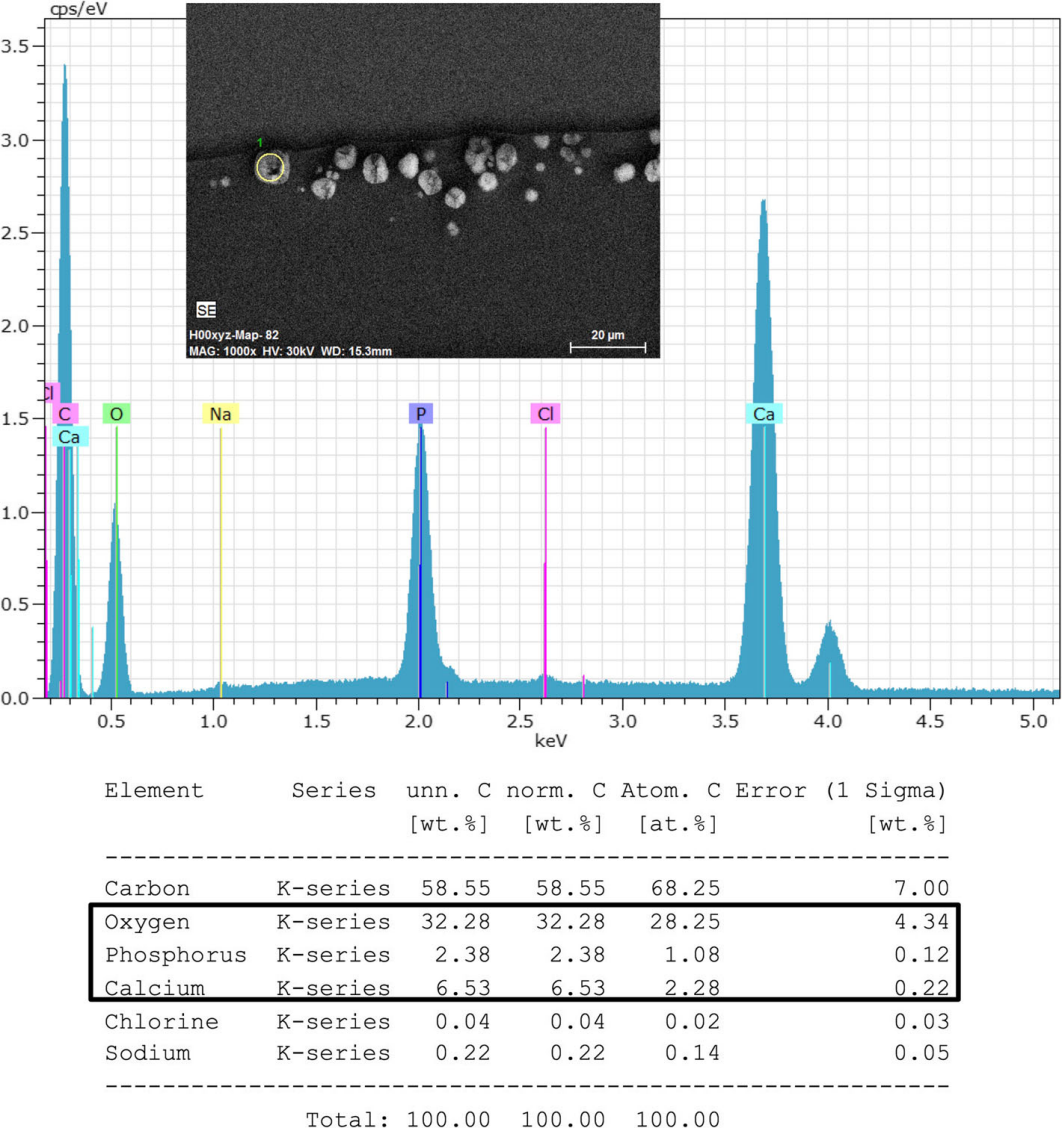
Exemplary elementary analysis (IOL 5) using energy dispersive X-ray spectroscopy showing the deposits to consist of calcium phosphate

SEM revealed numerous fine and roughly spherical crystalline deposits of approx. 2–15 μm in diameter that were situated immediately underneath the optical surface of the IOL, often causing micro-distortions of the optical surface (Fig. 3). In some cases there appeared to be microvacuoles within the crystals causing microscopically small cracks or openings through the optical surface (Fig. 3, IOLs 4 and 9). Element mapping (Fig. 4) and X-ray spectroscopy (Fig. 5) showed, that the deposits are made up of calcium phosphate.

**Fig. 6:**
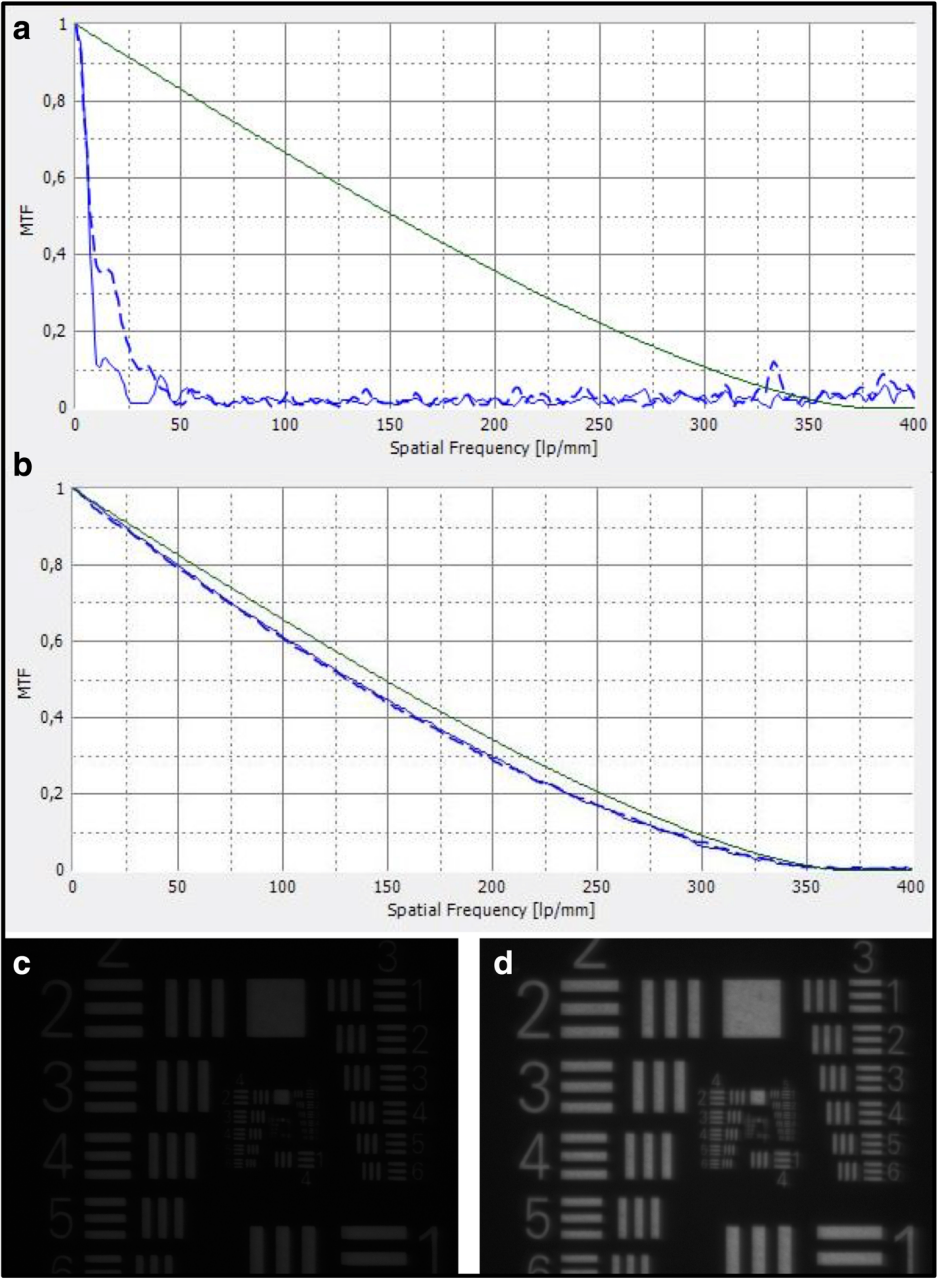
(**a**) Representative modulation transfer function (MTF) of an opacified IOL (IOL 6) showing a deterioration at all spatial frequencies. The green line represents the diffraction limit. Blue lines represent the measured MTF values in sagittal (solid blue line) and tangential (dashed blue line) planes. (**b**) Representative MTF of a clear monofocal aspheric IOL from hydrophilic acrylate (CT Asphina 409 MP, Carl Zeiss Meditec, Jena, Germany) taken in our laboratory for comparison. (**c**) USAF target image obtained through IOL 6 at standard lighting conditions showing a significant reduction in light transduction. (**d**) With light adjustment an acceptable image quality could be achieved

MTF measurements of those IOLs that were received with an intact optic (IOLs No. 2–4, 6–8 and 10–13) showed a significant decrease in optical quality with MTF values deteriorated at all spatial frequencies (Fig. 6a). The analysis of USAF target images illustrates a significant loss in brightness and contrast, however, with light adjustment an acceptable image resolution could be achieved (Fig. 6b,c).

## Discussion

Since the advent of minimally invasive keratoplastic surgery, particularly DS(A)EK and DMEK, there has been an increasing number of reports on postoperative opacification of hydrophilic acrylic IOLs [6–13, 15–18]. These techniques commonly rely on the instillation of an air or gas-bubble into the anterior chamber to foster the adhesion of the graft tissue with the posterior corneal surface [19, 20]. Whereas in previous reports on IOL opacifications not associated with secondary surgical interventions the whole optical surface (including the posterior surface) of the IOL and in some cases even the haptics showed signs of opacification [2, 3], suggesting material impurities or faulty manufacturing, the opacification pattern observed in hydrophilic IOLs after posterior lamellar keratoplasty is very distinct. The opacified area in these cases is usually limited to a more or less circular area of the anterior optical surface, which usually corresponds to the area of the pupil and/or anterior capsulorhexis, thus to the zone of contact with the instilled air or gas, whereas parts of the IOLs covered by the capsular bag, including the haptics and the posterior optical surface, displayed no sign of opacification [6–11, 13, 15, 16]. Furthermore, it appears to equally affect hydrophilic IOLs from different manufacturers (Bausch & Lomb [6, 13], Ciba Vision [11, 13], Lenstec [17], Oculentis [10, 13], Rayner [8, 17], Acri.Tec [16], Zeiss [18]). It seems, that the instillation of exogenous material into the anterior chamber and its prolonged direct contact to the IOL’s surface increases the risk of opacification of hydrophilic acrylic IOLs irrespective of the manufacturer and the exact material composition.

It should be noted at this point, that the high amount of Rayner IOLs in this study is predominantly owed to the circumstance, that Rayner Intraocular Lenses Ltd. routinely send their IOLs to our laboratory for pathological analysis, whereas IOLs from other manufacturers only reach us through individual cooperating ophthalmic surgeons. The many reports on IOL calcification after posterior lamellar keratoplasty clearly point to a general problem with the hydrophilic acrylate material, rather than a problem with IOLs from certain individual manufacturers.

To the best of our knowledge, this is the largest series of opacified IOLs after posterior lamellar keratoplasty analysed to date. Our results show, that the opacification of the analysed IOLs is caused by crystalline deposits underneath the anterior optical surface, which stain positively with Alizarin Red and the von Kossa method, suggesting a high calcium content. Energy dispersive X-ray spectroscopy for elementary analysis of the composition of the deposits proves, that the deposits consist of calcium phosphate. This confirms the findings of other groups, who demonstrated calcium phosphate deposits in explanted IOLs using similar methods [2, 3, 7, 10, 13, 18, 21].

The calcification-pattern occurring after posterior lamellar keratoplasty as described here and in other reports in the literature was also observed in procedures not involving air or gas instillation into the anterior chamber. Fung et al. report 7 cases of opacified hydrophilic IOLs after intracameral injection of rtPA to treat inflammatory fibrin membranes secondary to cataract surgery [5]. In 3 of these patients an IOL-exchange was performed. Similar to the results reported here, the laboratory analysis of the explanted IOLs showed calcium phosphate deposits on the surface/subsurface of the IOLs' anterior optical surface.

Different pathomechanisms have been suggested to play a role in hydrophilic IOL opacification after posterior lamellar keratoplasty and air or gas instillation into the anterior chamber. Metabolic changes in the anterior chamber due to the presence of the exogenous gas or other substances and an exacerbated inflammatory reaction caused by often multiple surgical procedures may lead to changes in the aqueous humor composition and cause the calcification of the IOL surface [13, 15, 18]. The supposed disturbance of the blood-aqueous-barrier caused by underlying conditions (mostly Fuchs’ endothelial dystrophy) may amplify metabolic imbalance and intraocular inflammatory processes [17, 18]. We believe, however, that the direct and prolonged contact of the IOL with the instilled exogenous gas, air or other substance is the key factor in the opacification of hydrophilic acrylic IOLs after posterior lamellar keratoplasty. The direct contact of the air or gas with the IOL possibly affects the IOL surface in a way that fosters the formation of crystallisation nuclei [10]. Dehydration of the hydrophilic acrylate material in contact with the exogenous air or gas may be one possible causative mechanism.

Especially in early stages of IOL-calcification, glistenings may be considered as an important differential diagnosis. In contrast to the opacifications in hydrophilic IOLs reported here, glistenings can occur in hydrophobic IOL materials. There are numerous reports in the literature on the formation of glistenings, especially in the AcrySof IOL (Alcon Laboratories, Fort Worth, Texas, USA) [2, 22, 23]. However, glistenings have also been observed in hydrophobic IOLs from other manufacturers [23]. Morphologically they correspond to microvacuoles within the hydrophobic IOL material. Although glistenings seem to reduce contrast sensitivity, a reduction of visual acuity is rare [23–26]. Aside from calcifications, other alterations of the IOL material have been described, such as IOL schisis, which is caused by the formation of a gap within the IOL material and may also reduce contrast sensitivity [27].

In addition to the laboratory analysis we assessed the optical quality of the calcified IOL explants by performing an optical bench analysis. This is the test method suggested in ISO 11–979-2 as the most suitable method for evaluating an IOLs optical properties. The ISO guidelines suggest that the system of model eye and IOL should exhibit an MTF value greater than 0.43 at 100 lp/mm. Our results clearly showed a deterioration of the optical quality with a marked decrease of the MTF across all spatial frequencies. Clinically this translates into a reduction of brightness and contrast especially in mesopic conditions and ultimately to „foggy “or „clouded “vision and loss of overall visual acuity. We have shown similar results in faulty manufactured IOLs in earlier publications [3, 21], but to our knowledge this is the first systematic examination of calcified IOL explants after posterior lamellar keratoplasty using an optical bench in a larger series.

In symptomatic patients with decreased visual acuity caused by IOL calcification, an IOL explantation is the only treatment option. Previously, the incidence of IOL opacification/calcification was reported to be less than 1% [2]. However, with the advent of microinvasive keratoplastic techniques, reports about IOL opacifications and subsequent explantations have increased. Ahad et al. reported an incidence of 9.7% IOL opacifications in 154 eyes after DSAEK [28]. Nieuwendaal et al. report an incidence of IOL opacification of 5% in 160 eyes after DSEK with a 2.5% incidence of IOL exchange [29]. The explantation of IOLs involves the risk of intra- and postoperative complications. In a series of 25 eyes with opacified Aqua-Sense IOLs (a single-piece IOL from a hydrophilic acrylic copolymer with a water content of 25% by Aaren Scientific, Ontario, California, USA) Dagres et al. reported a complication rate (zonular dehiscence, posterior capsular rupture, corneal decompensation) of 48% [14]. Especially a strong adhesion of the IOL to the capsular bag can make the explantation procedure challenging and in many cases an in-the-bag IOL implantation may not be possible. The indication for surgical removal of the opacified IOL therefore requires thorough preoperative diagnostic and informed patient consent and should only be considered in highly symptomatic patients. A Nd:YAG-Laser capsulotomy should not be performed in eyes with opacified IOLs, since it further increases the complication rate during an IOL exchange procedure. Obviously, a Nd:YAG-Laser treatment of the opacified IOL’s surface, as has been intended in some cases (e.g. [13, 29]), will not be able to clear the opacifications that are situated underneath the optical surface and might in fact induce additional damage and further attenuate the IOL’s optical quality.

## Conclusion

IOL opacification is a rare postoperative complication. However, the instillation of air, gas or rtPA into the anterior chamber increases the risk of calcification for hydrophilic acrylic IOLs. This complication seems to be irrespective of the manufacturer. Since IOL explantation is associated with an increased intraoperative complication rate, it is recommended to avoid the use of hydrophilic acrylic IOLs in patients with endothelial dystrophy that will likely require procedures involving the intracameral instillation of air or gas, such as DMEK or DS(A)EK.
